# Interconnection of post-transcriptional regulation: The RNA-binding protein Hfq is a novel target of the Lon protease in *Pseudomonas aeruginosa*

**DOI:** 10.1038/srep26811

**Published:** 2016-05-27

**Authors:** Lucía Fernández, Elena B. M. Breidenstein, Patrick K. Taylor, Manjeet Bains, César de la Fuente-Núñez, Yuan Fang, Leonard J. Foster, Robert E. W. Hancock

**Affiliations:** 1 Centre for Microbial Diseases and Immunity Research, University of British Columbia, 2259 Lower Mall, Vancouver BC, Canada; 2 University of British Columbia, Centre for High-Throughput Biology and Department of Biochemistry & Molecular Biology, Vancouver, BC, V6T 1Z4, Canada

## Abstract

Besides being a major opportunistic human pathogen, *Pseudomonas aeruginosa* can be found in a wide range of environments. This versatility is linked to complex regulation, which is achieved through the action of transcriptional regulators, and post-transcriptional regulation by intracellular proteases including Lon. Indeed, lon mutants in this species show defects in motility, biofilm formation, pathogenicity and fluoroquinolone resistance. Here, the proteomic approach stable isotope labeling by amino acids in cell culture (SILAC) was used to search for novel proteolytic targets. One of the proteins that accumulated in the lon mutant was the RNA-binding protein Hfq. Further experiments demonstrated the ability of Lon to degrade Hfq *in vitro*. Also, overexpression of the *hfq* gene in the wild-type strain led to partial inhibition of swarming, swimming and twitching motilities, indicating that Hfq accumulation could contribute to the phenotypes displayed by Lon mutants. Hfq overexpression also led to the upregulation of the small regulatory RNA PhrS. Analysis of the phenotypes of strains lacking or overexpressing this sRNA indicated that the Lon protease might be indirectly regulating the levels and activity of sRNAs via Hfq. Overall, this study revealed new links in the complex regulatory chain that controls multicellular behaviours in *P. aeruginosa*.

Bacteria have evolved sophisticated mechanisms to adapt to the abundant and diverse stresses present in their environment. These adaptations frequently involve changes in the proteome in response to external stimuli. This is partly achieved by means of transcriptional regulation, which ultimately leads to the production of different subsets of proteins. However, the protein profile of bacterial cells is also controlled through degradation mediated by intracellular proteases and through regulation of translation by small non-coding RNAs in conjunction with RNA-binding proteins. Indeed, the importance of intracellular proteases in the global regulation of cellular activities related to metabolism, stress, virulence and antibiotic resistance has become widely accepted. Thus, in addition to degrading misfolded or defective proteins, proteases can control the levels of stress-related proteins as well as labile regulators and chaperones^1^. One of these intracellular proteases is the ATP-dependent Lon protease that has been found in the genome of numerous microorganisms. Lon is a cytoplasmic serine protease that typically associates into hexameric rings in Gram-negative bacteria and belongs to the group of chambered or self-compartmentalized proteases. Unlike other ATP-dependent proteases, Lon is a homo-oligomer consisting of an N-terminal domain, an ATP-binding domain, a substrate sensor and discriminatory domain, and a proteolytically active C-terminal domain within the same peptide chain^2^. Lon has been well characterized in *Escherichia coli*. Lon together with the hetero-oligomer complex formed by ClpP account for approximately 70–80% of all intracellular energy-dependent proteolytic activity^3^. In *E. coli*, Lon is known to degrade abnormal proteins as well as physiological targets, including the antitermination (N) protein of phage λ^4^,^5^, the cell division inhibitor SulA^6^, and the capsule transcriptional activator RcsA^7^.

Recent studies indicate that the Lon protease also plays an important role in the Gram-negative bacterium *Pseudomonas aeruginosa*. This microorganism is a major opportunistic human pathogen, which is responsible for a high percentage of nosocomial infections in immunocompromised or burn patients, as well as one of the main bacteria that leads to morbidity and mortality in cystic fibrosis patients^8^,^9^. The significant role of intracellular proteases in controlling virulence, antibiotic resistance and stress responses of *P. aeruginosa* is being increasingly recognized^10^,^11^. In particular, the Lon protease is important for responses to DNA damage stress, resistance to DNA-targeted antibiotics, pathogenesis and virulence-related properties. For example, *lon* mutants show increased susceptibility to fluoroquinolones, due to the reduced ability of the cell to trigger a DNA-damage response in the absence of Lon^12^,^13^,^14^. Similarly *lon* mutants of *P. aeruginosa* show a classic elongated (filamentous) morphology^12^ that gives the gene its name in *E. coli*^1^. Subsequently, Breidenstein *et al.*^14^ showed that the formation of filaments is likely to be related to an accumulation of the cell division inhibitor SulA, a known target of Lon in *E. coli*^6^. Additionally, the *lon* gene is upregulated upon exposure to fluoroquinolones and aminoglycosides, and during swarming motility, and is involved in regulating complex adaptations such as swarming motility and biofilm formation^15^. Recently it was demonstrated that Lon had a direct involvement in pathogenesis in chronic and acute animal models^16^.

Here we set out to identify new Lon targets in *P. aeruginosa* by identifying the proteins that accumulate in a *lon* mutant using the stable isotope labeling by amino acids in cell culture (SILAC) proteomic approach. This technique led to the identification of several proteins that appeared overrepresented in the *lon* mutant compared to the wild type and, as such, were likely targets for degradation by the Lon protease. Our results indicate that the Lon protease might potentially be at the top of an intricate regulatory cascade controlling multiple phenotypes via post-transcriptional regulation by small non-coding sRNAs as a result of its proteolytic activity affecting the RNA-binding protein Hfq. This links two mechanism of post-transcriptional regulation whereby protease processing influenced translational regulation by sRNAs.

## Results and Discussion

### SILAC experiments

As an intracellular protease, the regulatory activity of Lon is expected to depend on the degradation of labile proteins. Therefore, it is necessary to identify the intracellular substrates of Lon to fully understand how the loss of this protease results in such a remarkable array of phenotypic changes. One way of screening for potential Lon targets is by comparing the proteome of a *lon* mutant to that of the wild-type strain in order to identify proteins overrepresented in the mutant. Indeed, SILAC has been useful in identifying new targets of intracellular proteases, such as ClpPX from *E. coli*^17^. In this study, a proteomic analysis was carried out following the SILAC approach, which was based on labeling two isogenic strains with different amino acid isotopes (“heavy”- versus “light”-labeled). These experiments were carried out using the strain *P. aeruginosa* H399, which was auxotrophic for leucine and lysine. Thus, a Lon deletion mutant in the H399 background was obtained by replacing the *lon* gene with a gentamicin resistance cassette (Materials and Methods). To prepare the samples for SILAC experiments, the H399 wild type and its *lon* deletion mutant were grown in BM2 minimal medium supplemented with lysine and leucine. However, in the case of the *lon* mutant, natural lysine was substituted with lysine labeled with a heavy isotope (LysD4). After growth, the cytoplasmic fraction from each strain was prepared as described below and then mixed in a 1:1 ratio prior to digestion with the protease LysC. The result of this was a mix of peptides that were purified, fractionated and finally used for mass spectrometry analysis. The different masses of the proteins of the two strains, heavy (H) for the mutant and light (L) for the parent, facilitated comparison between the two samples. SILAC was performed four independent times, and only proteins that showed differences in abundance in at least three repeats were considered for further analysis. To identify proteins susceptible to degradation by Lon protease, we focused on those proteins that showed greater levels in the mutant than in the parent [protein ratio heavy versus light (H/L) >2], leading to a fairly small number of proteins (Table 1). This could indicate that Lon only has a minor effect on the proteome of *P. aeruginosa*, but the results were distorted by the inability of the proteomic methods used to identify differences in low-abundance proteins, e.g. certain transcriptional regulators. Moreover, the proteins identified by this method were also limited by the specific growth conditions used in the experiment. Indeed, future experiments could aim to identify additional targets by using under alternative growth conditions. For instance, it would be interesting to identify proteins that accumulate following fluoroquinolone treatment, since Lon is known to affect the SOS response in *P. aeruginosa*^14^.

The proteins that accumulated in the *P. aeruginosa* Lon mutant belonged to several functional classes, including 3 hypothetical proteins, 5 proteins involved in metabolism, the chaperone GroEL, and the RNA-binding protein Hfq (Table 1). This multiplicity of potential targets is not surprising given the array of phenotypes exhibited by Lon mutants. However, it must be noted that accumulation of a protein in the Lon mutant did not definitively indicate its direct degradation by the Lon protease. Consequently, we directly assessed *in vitro* degradation of selected proteins by Lon.

### *In vitro* activity of Lon

**Figure 1:**
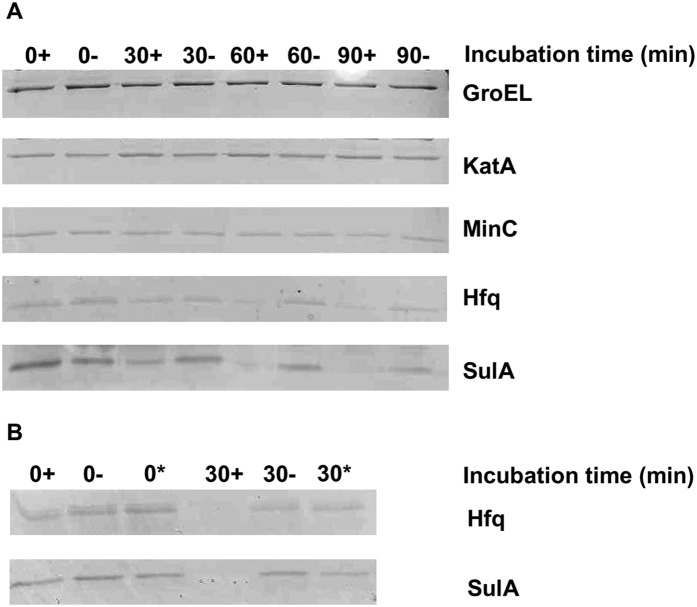
*In vitro* degradation of selected proteins by the Lon protease. His-tagged proteins (0.48 μM of GroEL, KatA, MinC, Hfq and SulA) were incubated at 37 °C for 0, 30, 60 or 90 minutes in reaction buffer with 0.6 μM His-Lon (**A**), with (+) or without (−) ATP. Degradation of SulA and Hfq was further confirmed by including a second negative control without Lon (*) incubated under the same conditions (**B**).

Amongst the proteins identified through SILAC, three were chosen to test as to whether they were actual substrates of Lon using an *in vitro* degradation assay. These proteins included GroEL and KatA, which are respectively involved in heat shock and oxidative stress, and the RNA-binding protein Hfq. Degradation of SulA was also investigated as a positive control, since it is known to be degraded by Lon in *E. coli*^6^ and is a likely Lon target in *P. aeruginosa*^14^, although SulA was not identified using our SILAC approach since it is inducible as part of the SOS response and minimally expressed under the growth conditions used. The candidate target proteins were purified and incubated with Lon and ATP at 37 °C for different amounts of time and then loaded onto an SDS-PAGE gel. As negative controls, the samples were also incubated without Lon, or without ATP that is essential for Lon activity. As expected, SulA was degraded to a greater extent in the sample incubated with Lon and ATP than in the two negative controls (Fig. 1A,B). Likewise, Hfq was degraded more readily when both ATP and the Lon protease were present during the incubation (Fig. 1A,B). In contrast, there was no significant difference between the test sample and the negative controls for GroEL or KatA, indicating that neither of these proteins was a direct Lon target under the experimental conditions (Fig. 1A).

Degradation of SulA by Lon had already been shown in *E. coli*^6^. However, in the case of *P. aeruginosa* degradation of SulA by Lon had thus far only been inferred from phenotypic evidence. Thus, overexpression of SulA in a wild-type background leads to some of the typical phenotypes observed in *lon* mutants, most notably cell filamentation^14^. The present study demonstrates that there is indeed a direct degradation of SulA by the Lon protease in *Pseudomonas*. In contrast to SulA, Hfq had not been previously described as a Lon target in any microorganism, although the current study opens up this possibility in other bacteria. Hfq, which participates in multiple stress response-related phenotypes in *E.coli*^18^, is a small protein that can bind a variety of sRNAs and enables them to bind to and regulate the translation of their target mRNAs, thereby exerting a post-transcriptional regulatory role that may have pleiotropic effects. In *P. aeruginosa*, previous studies had shown that Hfq is involved in virulence and it modulates the expression of quorum sensing signals^19^,^20^. Lon also appears to have an impact on quorum sensing in *Pseudomonas*^21^, and *lon* mutants exhibit phenotypes in quorum sensing-related phenomena such as biofilm formation and swarming motility^15^. Thus, the possible role, in these processes, of Hfq degradation by the Lon protease was studied in greater depth.

### Phenotypic effects of *hfq* overexpression and deletion

The gene encoding Hfq was cloned into the high copy vector pUCP18 and introduced into the wild-type strain *P. aeruginosa* PAO1 and an *hfq* deletion mutant. As a control, the same strains were transformed with the pUCP18 empty vector. Overexpression of *hfq* was confirmed by RT-qPCR analysis (data not shown).

**Figure 2:**
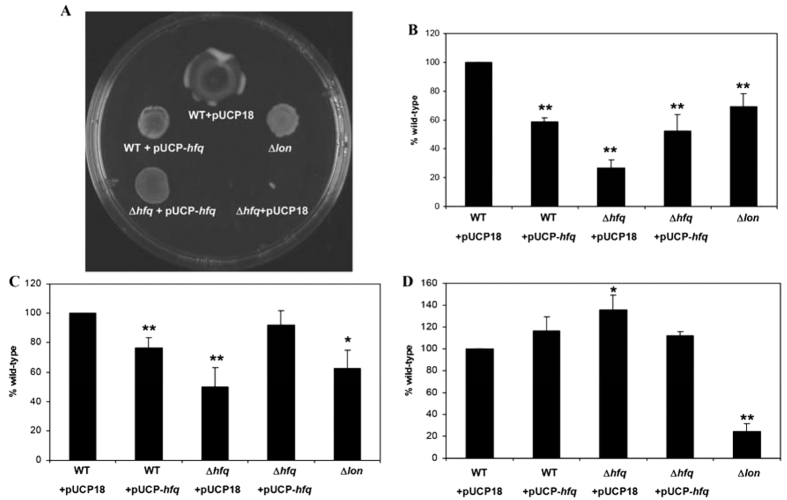
Impact of *hfq* overexpression on motility and biofilm formation. Swarming (**A**), swimming (**B**) and twitching (**C**) motility, as well as biofilm formation ability (**D**) were tested in the wild-type PAO1 strain (WT) and the *hfq* deletion mutant (Δ*hfq*) carrying plasmids pUCP18 (empty vector) or pUCP-*hfq* (vector for *hfq* overexpression), and the *lon* transposon mutant (Δ*lon*). A statistically significant difference according to the Student’s t test is indicated by ** or * for a P value of ≤0.03 or of <0.05, respectively.

The impact of Hfq on different types of motility, namely swimming, swarming and twitching, was assessed and compared to the phenotypes of the *lon* mutant strain. The results showed that *hfq* overexpression led to some degree of inhibition of the three motility types tested, resembling the inhibition displayed in the *lon* mutant in which Hfq would not be degraded and hence would be more abundant (Fig. 2A–C). Statistical analysis of the swimming and twitching motility results confirmed that no significant differences could be observed between the WT strain overexpressing *hfq* and the *lon* mutant (P values > 0.05), and that both strains had defects in both motilities compared to the wild type (Fig. 2B,C). Deletion of the *hfq* gene had an even greater effect on motility that could be partially complemented by introducing the gene in trans (Fig. 2A–C). These results indicate that both the excess and the lack of the Hfq protein had a negative impact on motility in *Pseudomonas*. Interestingly, similar observations were made with regards to the effects of the loss or overexpression of the Lon protease. The overexpression of *lon* in the wild-type PAO1 strain partially inhibited swarming motility^22^ while its deletion due to mutation led to complete loss of swarming motility^15^. Neither the strain overexpressing *hfq* nor the *hfq* deletion mutant displayed a reduced ability to form biofilms under the assayed conditions, unlike the biofilm-deficient *lon* mutant (Fig. 2D). Therefore, it seems that the biofilm phenotype observed in *lon* mutants might be due to the effect of Lon on an as-yet unidentified target, e.g. quorum sensing^21^. In the future, the identification of this target might be facilitated by performing SILAC on biofilm cells.

The accumulation of Hfq in *lon* mutants might explain why the main catalase, KatA, was overrepresented in the Lon mutant even though it does not seem to be directly degraded by this protease. Indeed, Sonnleitner *et al.*^20^ observed that Hfq mutants produced less catalase activity. Additionally, increased production of KatA is a known mechanism of response to the oxidatively active compound pyocyanin^23^, and pyocyanin hyperproduction has been shown to result from *hfq* overexpression (data not shown).

As mentioned above, *Pseudomonas lon* mutants exhibit increased susceptibility to fluoroquinolones. However, neither deletion nor overexpression of the gene encoding Hfq had any effect on ciprofloxacin susceptibility (data not shown). Likewise, overexpression of *hfq* did not lead to cell filamentation, a typical phenotype observed in Lon mutants, which is known to be related to the accumulation of SulA (data not shown). Taken together, these results indicate that additional Lon targets are likely to be responsible for the biofilm defect and the ciprofloxacin susceptibility phenotypes.

### Dysregulation of the small non-coding RNA *phrS* by overexpression of Hfq

As mentioned above, Hfq plays a key regulatory role by intermediating the activity of sRNAs. For this reason, we hypothesized that the effects observed in the *hfq* mutant and overexpression strains and, perhaps, in the *lon* mutant could be linked to changes in the activity of sRNAs. More specifically, we focused on the sRNA PhrS since it has been shown to bind to Hfq^24^,^25^.

To determine whether Hfq was acting via this sRNA, we first analyzed the expression levels of PhrS in response to *hfq* overexpression by RT-qPCR. These experiments showed that PhrS was upregulated by 5.93 ± 1.39 fold in the strain overexpressing *hfq* compared to the strain carrying the empty vector. The greater abundance of PhrS due to accumulation of Hfq in the Lon mutant was consistent with the accumulation of GroEL in the *lon* mutant, despite GroEL not being a direct substrate of this protease. Indeed, this is consistent with the demonstration^24^ that overexpression of *phrS* in *P. aeruginosa* led to a higher level of GroEL.

### Phenotypic effects of *phrS* overexpression and deletion

**Figure 3:**
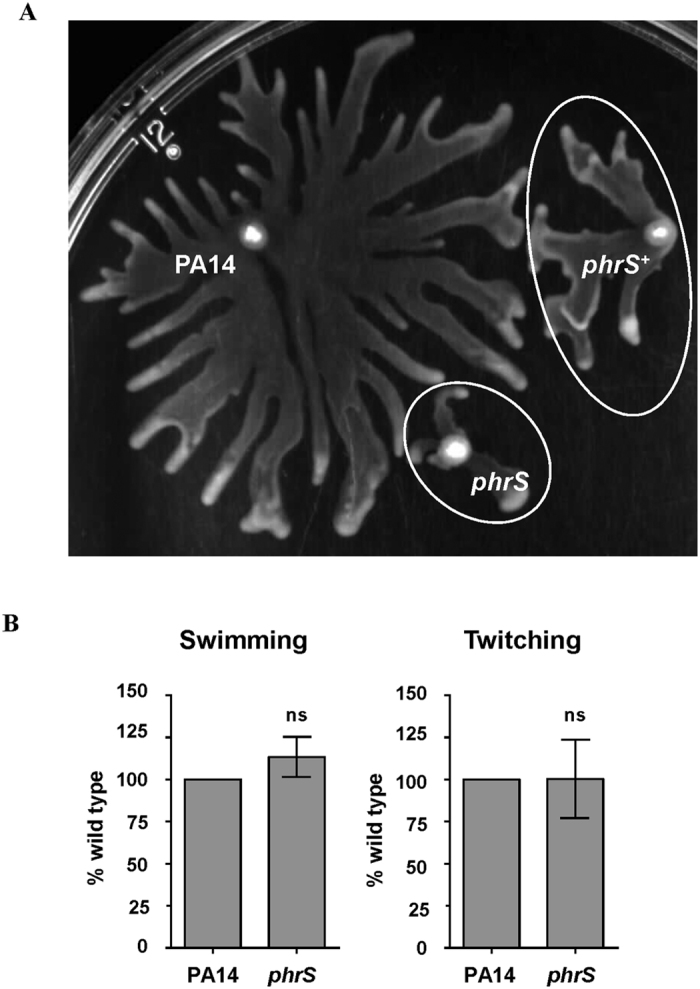
Impact of *phrS* deletion and overexpression on motility. Swarming motility of wildtype PA14, *phrS*, and *phrS*^+^ complemented mutant (**A**). Areas covered by the mutant and the complemented strain are marked by a circle to facilitate comparison to the wild type. Please note that the swarming clone morphology of strain PA14 (dendritic appearance) is quite different from that of strain PAO1 and derivatives (shown in Fig. 2). Mid-log growth cultures grown in BM2-glucose were inoculated on BM2-glucose swarm plates lacking (NH_4_)_2_SO_4_ and incubated for 18 h at 37 °C. Shown is a representative plate of three biological replicates. Swimming and twitching motility of wildtype PA14 and *phrS* (**B**). Swim and twitch plates were inoculated then incubated for 18 h at 37 °C and 72 h at room temperature, respectively. Error bars represent the standard error of the mean for three biological replicates. An unpaired Student’s t test was used to determine there was no significance (ns) between wildtype PA14 and *phrS* mutant strains.

Having established that both Lon and Hfq participated in the complex regulation of swarming motility in *P. aeruginosa*, we sought to determine if the lack or excess of the sRNA PhrS could also affect swarming. The *phrS* mutant displayed a major swarming defect compared to the wild-type strain (Fig. 3A) and this defect was partially restored in the complemented strain (*phrS*^+^). One possible explanation for only achieving partial complementation was that overexpression of *phrS* also had an effect on swarming motility, as observed for both *lon* and *hfq* (Fig. 3A). In contrast to results for swarming, the *phrS* mutant showed no significant difference in either swimming or twitching motility compared to the wild type (Fig. 3B). Therefore, PhrS had an effect on swarming motility but did not appear to affect the expression of functional flagella or type IV pili indicating that it controls some other process involved in induction of swarming (with more than 233 candidate genes; 26). Intriguingly, when we examined complementarity to PhrS by blast searching, we identified PA5378 as a target. This gene encodes a putative periplasmic component of a glycine betaine/L-proline ABC transporter, and is essential for swarming motility^26^ and thus could be the direct target of PhrS that influences swarming.

**Figure 4:**
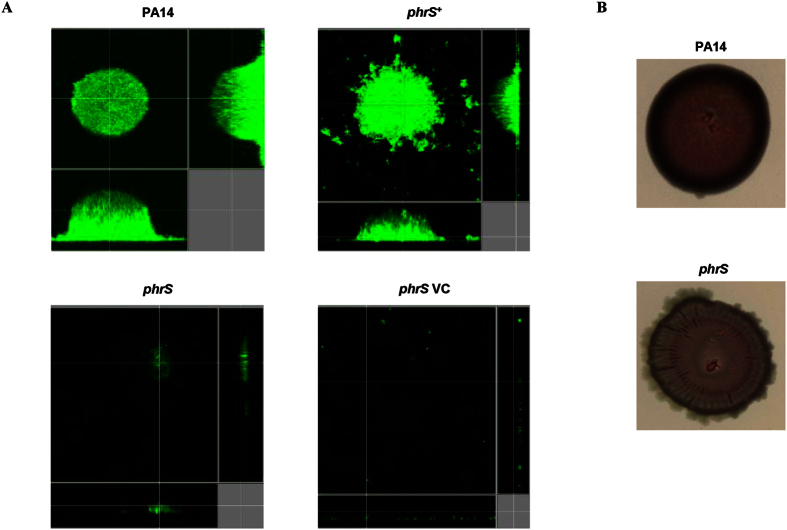
Impact of *phrS* deletion and overexpression on biofilm formation. (**A**) Flow cell chamber biofilm formation analysis of wildtype PA14, *phrS*, *phrS*^+^ complemented mutant, and *phrS* VC strain carrying the empty plasmid. Mid-log cultures were inoculated and then incubated in BM2-glucose for 72 h at 37 °C. Biofilm cultures were stained with SYTO-9 and visualized by confocal laser-scanning microscopy. Scale bars indicate 15 μm. Each panel is represented in xy, yz, and xz dimensions. (**B**) Congo red staining of wildtype PA14 and *phrS*. Strains were cultured overnight in tryptone broth before aliquots were inoculated on Congo red agar plates and incubated for 5 days at room temperature. Shown is one representative plate of three biological replicates.

The effect of PhrS deletion or overexpression on biofilm formation was also tested in a flow cell system. The *phrS* mutant displayed a highly reduced ability to develop a biofilm compared to the wild-type strain PA14 (Fig. 4A). Complementation of *phrS* in trans led to complete restoration of wild-type levels of biofilm formation (Fig. 4A). A *phrS* mutant strain (*phrS* VC) carrying the empty pUCP18 expression vector was used to confirm that the plasmid itself was not contributing to the biofilm deficient phenotype observed (Fig. 4A). In addition to flow cell chamber analysis, Congo red staining for the polysaccharides secreted by biofilm colonies (particularly the polysaccharide synthesized by the products of the *pel* operon) was performed. In contrast to the drastic reduction in biofilm formation observed in flow cell chambers, the *phrS* mutant displayed only a moderate decrease in Congo red staining compared to the wild type (Fig. 4B). This indicates that the defect in biofilm formation of this strain was due to factors other than reduced polysaccharide production. Indeed, these data suggest that PhrS might be involved in the regulatory pathways that lead to the development and maturation of biofilms. PhrS controls the post-transcriptional production of PqsR that itself transcriptionally regulates the production of the *Pseudomonas* quinolone signal PQS, a component of a quorum sensing system^27^,^28^,^29^. In this regard, it has been shown that the disruption of PQS production in *P. aeruginosa* causes major changes to the morphology of biofilms^30^. There are also other examples of sRNAs having regulatory roles in biofilm formation such as the extensively studied RsmY and RsmZ pair of sRNAs acting through RNA-binding protein RsmA^31^ and *crcZ* acting through RNA-binding protein Crc^32^.

Swarming motility and biofilm formation are often considered to represent the dichotomy between acute versus chronic infectious disease states. Nonetheless, these two growth states in *P. aeruginosa* are coordinately regulated by Lon^15^ and this appeared to be mediated at least in part through Hfq and PhrS. Importantly, we have demonstrated here a link between two types of post transcriptional regulation whereby an intracellular protease regulates the amount of an RNA-binding protein that collaborates with an sRNA, PhrS, to influence two complex lifestyle adaptations, swarming motility and biofilm formation.

## Materials and Methods

### Strains, plasmids and growth conditions

The strains and plasmids used in this study are shown in Table 1 & 2. *E. coli* and *P. aeruginosa* strains were routinely grown in Luria-Bertani broth (LB) unless stated otherwise. When necessary the following antibiotic concentrations were added to the growth medium: gentamicin, 30 μg/ml; ampicillin, 100 μg/ml; carbenicillin, 500 μg/ml; streptomycin, 200 μg/ml; kanamycin, 50 μg/ml. All antibiotics were purchased from Sigma.

### Construction of an auxotrophic Lon deletion mutant

To carry out the SILAC approach, it was first necessary to construct a *lon* knock-out strain in an auxotrophic background. In this case, the strain used was *P. aeruginosa* H399, which is auxotrophic for the amino acids leucine and lysine. The plasmid construct necessary for replacing the *lon* gene by a gentamicin cassette was obtained by 3-step-fusion PCR^33^. Primers were designed to amplify the upstream and downstream regions of the *lon* gene as well as the gentamicin resistance gene from the pPS858 plasmid by PCR. Of note, some of these primers carried an overlapping sequence that allowed the sequential fusion of the three fragments in consecutive PCR reactions. The so-obtained fragment was then cloned into pCR-Blunt II-TOPO (Invitrogen) and later subcloned into the suicide vector pEX18Amp to generate plasmid pEX18-Δ*lon*. This plasmid construct was then transferred from *E. coli* S17-1λ*pir* into *P. aeruginosa* H399 by conjugation. The transconjugants were selected on plates with gentamicin and sucrose in order to promote homologous recombination and loss of the cointegrate. Deletion of *lon* was confirmed by PCR and subsequent sequencing.

### Growth of the wild-type and *lon* deletion mutant strains with stable isotopic labeling and preparation of cytoplasmic fraction

The auxotrophic wild-type H399 and its isogenic *lon* mutant strain were grown overnight at 37 °C in BM2-succinate medium [62 mM potassium phosphate buffer (pH 7), 7 mM (NH_4_)_2_SO_4_, 10 μM FeSO_4_, 20 mM succinate, 2 mM MgSO_4_] supplemented with the amino acids leucine and lysine at 52 mg/l and 72.5 mg/l, respectively. Both strains were grown with natural leucine; however, deuterium-labeled lysine (“heavy D4”) was used instead of the normal isotope (“light”) amino acid in the case of the *lon* mutant. To extract a cytoplasmic fraction, 10 ml of each culture was pelleted and washed with a buffer containing 50 mM Tris (pH 8.0) and 0.2 M MgCl_2_. Following this washing step, the samples were incubated for 30 minutes at 30 °C in the presence of the HALT protease inhibitor (Sigma-Aldrich) and subsequently chilled in an ice-water bath for 5 minutes and for 15 minutes at room temperature. The cells were pelleted and later washed with 50 mM Tris, resuspended in the same buffer and treated with 100 μg/ml DNase I for 20 minutes at 4 °C. Then, cell lysis was accomplished by sonicating the cell suspension six times for 10 seconds with a probe sonicator (Fisher) with 10 second intervals between pulses. The unlysed cells were removed by centrifugation at 100,000 × g for 1 h in a Beckman Coulter ultracentrifuge. The supernatant containing the cytoplasmic fraction was collected for use in the SILAC approach.

### SILAC experiments

The differences in the proteome between the wild type and the *lon* mutant were evaluated by utilizing the SILAC technique. The cytoplasmic fractions from both strains were extracted as described above and their protein content was quantified using the Bradford assay (Oz Bioscience). Then, 50 to 100 μg/ml of the supernatants corresponding to the parent and the *lon* mutant strains, containing light or heavy-labeled proteins respectively, were mixed together and then treated with In-solution-digest plus sodium deoxycholate. Briefly, 3% deoxycholate was added to the samples, which were then boiled for 5 min. Then the samples were incubated overnight at 37 °C with iodoacetamide and LysC, an endoproteinase that hydrolyzes at the carboxyl side of lysine. The peptides obtained in this digestion were purified following the C18 (desalted column) STAGE-Tip purification protocol. To prepare for binding, methanol was first added to the column and the peptide sample was loaded onto the column after dilution in a buffer containing 3% acetonitrile, 1% trifluoroacetic acid and 0.5% acetic acid. The sample was then eluted from the column with a buffer containing 0.5% acetic acid and 80% acetonitrile. Fractionation of the sample was carried out by isoelectric focusing with an OFFGel Fractionator (Agilent). The resulting fractions underwent a second C18 STAGE-Tip purification process prior to mass spectrometry (MS) analysis on a LTQ-OrbitrapXL (Thermo), as described^34^. Once collected, the raw data files were analyzed using the program MaxQuant^35^ for the detection of peak features and peptide quantification. Then, the spectra were associated with specific amino acid composition by using the program Mascot^36^ and the identified peptides linked with known protein sequences of *P. aeruginosa*, and then assembled into proteins. The data obtained permitted the calculation of the ratio between the levels of a given protein in the *lon* mutant (heavy labeled) and the parent strain (light labeled).

### Purification of the Lon protease and the candidate target proteins

To purify the Lon protease and candidate target proteins of interest, primers were designed to clone the respective genes into the pET28a vector to obtain N-terminally hexa-histidine (His_6_)-tagged proteins. The constructs were transformed by electroporation into *E. coli* BL21 cells that were grown for 2 h at 37 °C, followed by induction with IPTG for another 4 h at the same temperature. The cells were then harvested and resuspended in 5 ml of sonication buffer (500 mM NaCl, 5 mM MgCl_2_, 50 mM NaPO_4_ pH 7.8) containing 10 mM imidazole and subsequently lysed by sonication on ice (4 30-sec treatments). After sonication, cell debris and unbroken cells were pelletted by centrifugation at 8,000 rpm for 10 min. The supernatant was collected in a fresh tube and gently shaken for 1 h at 4 °C in the presence of 2 ml Ni-NTA resin (Qiagen). After that, this mix was poured into a Biorad disposable column and the proteins were eluted with sonication buffer containing 200 mM imidazole and 5% glycerol. The proteins were then run on an SDS-PAGE gel to assess their purity. The eluted proteins were dialyzed with a buffer containing 50 mM Tris-HCl pH8, 250 mM NaCl, 5mM DTT and 5% glycerol^37^.

### *In vitro* assay for degradation of the target proteins by Lon

The ability of His_6_-Lon to degrade the selected candidate target proteins (His_6_-SulA, His_6_-GroEL, His_6_-KatA and His_6_-HFQ) was tested by incubating 0.6 μM of His_6_-Lon and 0.48 μM of the target His-tagged proteins at 37 °C in a buffer containing 50 mM Tris-HCl (pH 8.0), 7.5 mM MgCl_2_ and 4 mM ATP as described previously^37^. In parallel, negative controls without ATP, which is necessary for Lon activity, or without Lon, were performed under the same conditions. At specific times, aliquots were taken from each sample, mixed with an equal volume of 2x loading buffer and boiled for 10 minutes prior to SDS-PAGE analysis. The gels were stained with Coomassie brilliant blue (His_6_-SulA, His_6_-GroEL and His_6_-KatA) or silver staining for small proteins (His_6_-Hfq). The assays were repeated independently at least three times.

### Overexpression of *hfq*

The Hfq-encoding gene was amplified from the *P. aeruginosa* PAO1 genomic DNA by PCR using the following primers: Chfq-F, CCAGATGCTGGAACAGGGTT; and Chfq-R, CCTTGCCACTGCCGATAAGA, which were designed using program Primer3^38^. The resulting amplicon was cloned into Zero-Blunt TOPO vector (Invitrogen) and then subcloned into pUCP18, thereby generating plasmid pUCP18-*hfq*. Plasmids pUCP18 and pUCP18-*hfq* were subsequently transformed into the *P. aeruginosa* PAO1 wild-type strain and the *hfq* deletion mutant^20^ as described previously^39^.

### Motility and biofilm-formation assays

Swimming and swarming motility were, respectively, tested on plates containing BM2-glucose with 0.25% (wt/vol) agar and BM2-glucose containing 0.5% (wt/vol) agar and 0.5% or 0.1% (wt/vol) casamino acids for strains PAO1 and PA14, respectively, instead of (NH_4_)_2_SO_4_^40^. Twitching was analyzed on LB plates containing 1.5% (wt/vol) agar. The degree of motility was determined after 24 hours of incubation at 37 °C.

Biofilm formation was assessed by crystal violet staining. Briefly, overnight cultures were diluted 1:100 into fresh LB and 100 μl aliquots were inoculated into each well of a microtiter plate. Following incubation at 37 °C for 24 h, the planktonic cells were removed and the attached cells were stained with 0.1% crystal violet for 20 min. Excess crystal violet was removed by washing 3 times with distilled water and then the dye was solubilized with ethanol for 20 minutes. The absorbance at 595 nm was measured to quantify biofilm formation.

### Analysis of gene expression by RT-qPCR

Three independent cultures of each strain were grown to the mid-logarithmic growth phase in LB, and total RNA was then isolated using RNeasy Mini kits (Qiagen). DNase treatment of RNA samples, cDNA synthesis, and real-time PCR (qPCR) were performed as described previously^41^. cDNA was diluted 1:100, and 2.5 μl were used as a template for each reaction, using 1× SYBR green PCR master mix (Applied Biosystems, Foster, CA). The reaction was carried out in an ABI Prism 7000 instrument (Applied Biosystems). Internal forward and reverse primers for each gene were designed using PrimerExpress (Applied Biosystems). Each sample was tested in duplicate. The fold-change was calculated following the Ct method and using the *rpsL* gene, encoding the 30S ribosomal protein S12, as a housekeeping gene.

### Complementation of the *phrS* mutant strain

A *P. aeruginosa* PA14 *phrS* mutant was utilized due to its availability and the generally similar swarming abilities of PAO1 and PA14 mutants. To complement the *phrS* mutant, the *phrS* gene locus was first amplified by PCR using the following primers: forward, 5′CTTGATGGCGAACTTGAGCG and reverse, 5′TTTGAACCTGACCTTCCGCC. The *phrS* amplification product was then ligated into a pCR-BLUNT II-TOPO cloning vector and transfected into *Escherichia coli* TOP10 strain using the TOPO PCR cloning kit from Life Technologies according to the manufacturer’s instructions. The *phrS* region was then ligated into the pUCP18 expression vector and transformed into *P. aeruginosa* by electroporation^39^. Confirmation of *phrS* expression was done by RT-qPCR.

### Biofilm growth in flow cell chambers

Sterilization and preparation of flow cell chambers, as well as growth of biofilms were carried out as previously described^42^,^43^. Briefly, mid-logarithmic cultures of the different strains were injected into sterile tubing with a syringe and a needle and left to adhere and replicate for 3 hours in static BM2-glucose media. Biofilms were then grown for 72 h in BM2-glucose pumped through the flow cell chambers at a rate of 2.4 ml/h by a Watson Marlow 205S peristaltic pump. Microscopy and imaging of biofilm microcolonies was performed as previously described^42^,^43^. An Olympus Fluoview FV1000 microscope was used to perform confocal laser-scanning microscopy. Biofilm cultures were stained with the all bacterial dye SYTO-9. Renderings of confocal images were made using the Imaris software package.

### Congo red staining

Congo red agar plates consisted of 1% (wt/vol) tryptone, 40 μg/ml Congo red, 20 μg/ml Coomassie blue, and 1% (wt/vol) agar. Overnight cultures were grown in 1% (wt/vol) tryptone broth and 10 μl were inoculated onto Congo red agar plates. The inoculated plates were incubated for 5 days at room temperature before being photographed.

### Concluding Remarks

Recent studies have shown the importance of intracellular proteases in controlling diverse phenotypes in *P. aeruginosa*, including pathogenicity, motility, biofilm formation and antibiotic resistance. However, there is not much information yet regarding their cytoplasmic protein targets that actually mediate the observed phenotypes. Here, despite the limitations of the SILAC approach, several potential targets of the Lon protease from *P. aeruginosa* could be identified. Three of these proteins were selected for further analysis due to their interest as putative regulators, ultimately revealing that the small protein Hfq is degraded by Lon. In addition to validating this method for identifying new proteolytic targets, this is very interesting due to the regulatory role exerted by Hfq inside the bacterial cell. Indeed, accumulation of Hfq due to overexpression conferred similar motility phenotypes to the deletion of Lon. In contrast, *hfq* overexpression did not appear to be related to other phenotypes observed in Lon mutants, such as filamentation, deficient biofilm formation and increased susceptibility. This clearly shows that proteases exert their regulatory function by modulating the intracellular levels of several proteins. As a result, it would be very interesting to carry out further experiments to identify additional Lon targets.

Overall, this study demonstrated for the first time that Lon participates in the degradation of two intracellular proteins, SulA and Hfq. Although SulA was already thought to be a target of Lon in *Pseudomonas*, Hfq was identified here through the SILAC method. Additionally, this work confirms that the sRNA PhrS, which usually works in conjunction with Hfq, is involved in the regulation of diverse processes in the Gram-negative pathogen *P. aeruginosa*, including social behaviors like biofilm formation and swarming motility.

## Additional Information

**How to cite this article**: Fernández, L. *et al.* Interconnection of post-transcriptional regulation: The RNA-binding protein Hfq is a novel target of the Lon protease in *Pseudomonas aeruginosa. Sci. Rep.* **6**, 26811; doi: 10.1038/srep26811 (2016).

## Figures and Tables

**Table 1:** Proteins overrepresented in the Lon mutant identified by SILAC.

PA number	Gene name	Gene description	Functional class^a^	Ratio H/L^b^
PA1673		Hypothetical protein	Hypothetical	5.9
PA1880		Probable oxidoreductase	Putative enzymes	4.3
PA3120	*leuD*	3-isopropylmalate dehydratase small subunit	Amino acid biosynthesis	3.2
PA4063		Hypothetical protein	Hypothetical	2.6
PA4236	*katA*	Catalase	Adaptation, protection	2.8
PA4333		Probable fumarase	Energy metabolism	2.6
PA4385	*groEL*	GroEL protein	Chaperone & heat shock proteins	2.7
PA4944	*hfq*	Hfq	Transcription, RNA processing degradation	2.3
PA5078		Conserved hypothetical protein	Hypothetical	2.6
PA5429	*aspA*	Aspartate ammonia-lyase	Amino acid biosynthesis	4.4

^a^Functional class taken from www.pseudomonas.com^44^.

^b^Indicates the average ratio between the heavy-labelled and the non-labelled (light) protein samples from 3 biological repeats.

**Table 2:** Bacterial strains and plasmids used in this study.

Strain or plasmid	Genotype or characteristics^a^	Reference
Strains		
*P. aeruginosa*		
H399	PAO1 auxotroph for leucine and lysine	45
Δ*lon*:H399	*lon* deletion in H399 background	This study
H103	Wild-type strain	Lab collection
PAO1*hfq*^−^	PAO1 *hfq::aadA*, Sm/Sp^r^	20
PAO1*lon*^−^	Transposon insertion mutant	46
PA14	Wild-type *P. aeruginosa* PA14	47
*phrS*	PA14 *phrS*::MrT7; Gm^r^	47
*phrS*^*+*^	*phrS*/pUCP18:*phrS*; Gm^r^, Ap^r^	This study
*E. coli*		
S17-1λ*pir*	λ(*pir*) *hsdR pro thi* RP4-2 Tc::Mu Km::Tn7	48
TOP10	F– *mcrA* Δ(*mrr*-*hsdRMS*-*mcrBC*) ϕ80*lacZ*M15 Δ*lacX74 recA1 ara*Δ*139* Δ(*ara*-*leu*)*7697 galU galK rpsL* (Str^r^) *endA1 nupG*	Invitrogen
Plasmids		
pCR-Blunt II-TOPO	PCR cloning vector; Km^r^	Invitrogen
pEX18Amp	Suicide plasmid; Ap^r^	49
pEX18-Δ*lon*	pEX18Amp carrying the fragment with the regions upstream and downstream of *lon* flanking a Gm resistance gene from pPS858	This study
pPS858	Source of the gentamicin cassette (Ap^r^, Gm^r^)	49
pET28a	Vector for His_6_-tagged protein overexpression (Km^r^)	Invitrogen
pET28a:*sulA*	His_6_-*sulA* cloned into pET28a	This study
pET28a:*hfq*	His_6_-*hfq* cloned into pET28a	This study
pET28a:*groEL*	His_6_-*groEL* cloned into pET28a	This study
pET28a:*katA*	His_6_-*katA* cloned into pET28a	This study
pUCP18	Vector for expression in *E. coli* and *P. aeruginosa*; Ap^r^	50
pUCP-*hfq*	*hfq* cloned into pUCP18	This study
pUCP-*phrS*	*phrS* cloned into pUCP18	This study

^a^Antibiotic resistance phenotypes: Ap^r^, ampicillin for *E. coli* and carbenicillin for *P. aeruginosa*; Ap^r^, ampicillin; Gm^r^, gentamicin; Km^r^, kanamycin; Sm/Sp^r^, streptomycin/spectinomycin; Tc^r^, tetracycline.
